# Multiresolution nondestructive 3D pathology of whole lymph nodes for breast cancer staging

**DOI:** 10.1117/1.JBO.27.3.036501

**Published:** 2022-03-21

**Authors:** Lindsey A. Barner, Adam K. Glaser, Chenyi Mao, Etsuo A. Susaki, Joshua C. Vaughan, Suzanne M. Dintzis, Jonathan T. C. Liu

**Affiliations:** a University of Washington, Department of Mechanical Engineering, Seattle, Washington, United States; b University of Washington, Department of Chemistry, Seattle, Washington, United States; c Juntendo University Graduate School of Medicine, Department of Biochemistry and Systems Biomedicine, Tokyo, Japan; d RIKEN Center for Biosystems Dynamics Research, Laboratory for Synthetic Biology, Osaka, Japan; e University of Washington, Department of Physiology and Biophysics, Seattle, Washington, United States; f University of Washington School of Medicine, Department of Laboratory Medicine and Pathology, Seattle, Washington, United States; g University of Washington, Department of Bioengineering, Seattle, Washington, United States

**Keywords:** open-top light-sheet microscopy, three-dimensional pathology, breast cancer, lymph node staging

## Abstract

**Significance:**

For breast cancer patients, the extent of regional lymph node (LN) metastasis influences the decision to remove all axillary LNs. Metastases are currently identified and classified with visual analysis of a few thin tissue sections with conventional histology that may underrepresent the extent of metastases.

**Aim:**

We sought to enable nondestructive three-dimensional (3D) pathology of human axillary LNs and to develop a practical workflow for LN staging with our method. We also sought to evaluate whether 3D pathology improves staging accuracy in comparison to two-dimensional (2D) histology.

**Approach:**

We developed a method to fluorescently stain and optically clear LN specimens for comprehensive imaging with multiresolution open-top light-sheet microscopy. We present an efficient imaging and data-processing workflow for rapid evaluation of H&E-like datasets in 3D, with low-resolution screening to identify potential metastases followed by high-resolution localized imaging to confirm malignancy.

**Results:**

We simulate LN staging with 3D and 2D pathology datasets from 10 metastatic nodes, showing that 2D pathology consistently underestimates metastasis size, including instances in which 3D pathology would lead to upstaging of the metastasis with important implications on clinical treatment.

**Conclusions:**

Our 3D pathology method may improve clinical management for breast cancer patients by improving staging accuracy of LN metastases.

## 1 Introduction

For the 300,000 patients who are diagnosed with invasive breast cancer or carcinoma *in situ* each year in the United States, the primary treatment method is lumpectomy (breast-conserving surgery) or mastectomy (breast-removal surgery). In addition to removal of the primary tumor, sentinel lymph nodes (LNs) are typically resected during the procedure to facilitate LN staging.^2^ The extent of LN metastases is widely regarded as the most important prognostic factor for breast cancer, in part because the spread of cancer to nearby nodes often precedes metastasis to distant organs.^1^ Sentinel LNs are evaluated with two-dimensional (2D) histology, in which metastatic lesions are classified based on their largest dimension. Metastases may be classified as isolated tumor cells (ITCs; <200  μm in diameter or a cluster of fewer than 200 cells), micrometastases (>200  μm in diameter but <2  mm in diameter), or macrometastases (>2  mm in diameter).^2^ The classification of each metastatic lesion determines the assigned LN stage, which indicates the overall status of LN metastases (if present). Typically, if macrometastases are identified (corresponding to stage pN1), patients are treated with complete axillary LN dissection (removal of all axillary nodes).

**Fig. 1:**
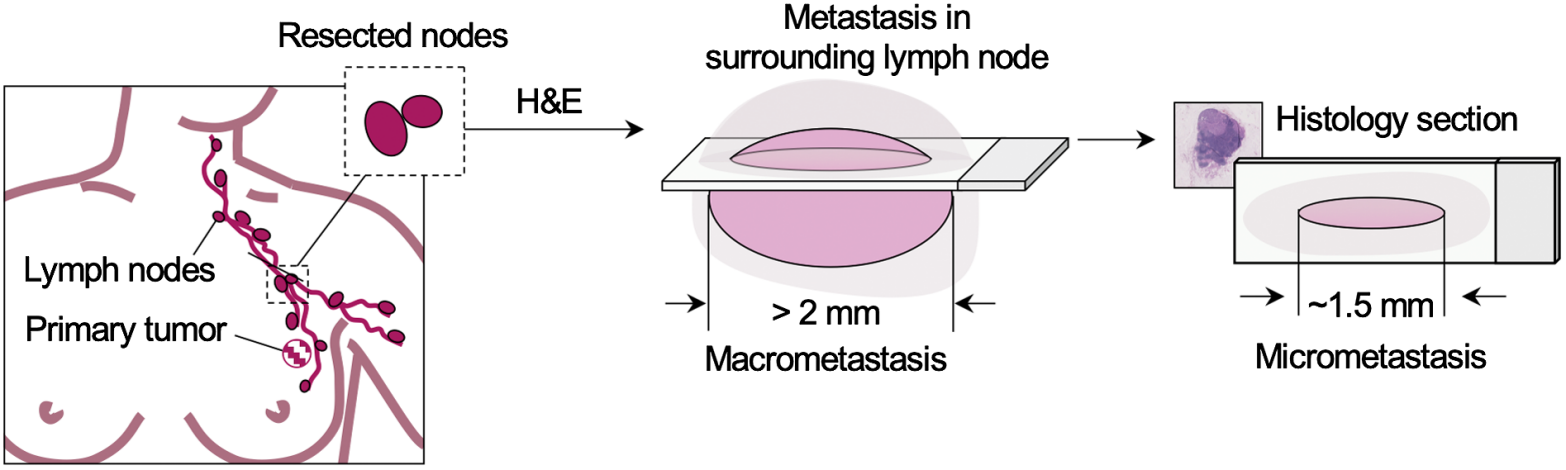
Sentinel LNs are resected during mastectomy or lumpectomy to facilitate evaluation of nodal metastases. However, sparse sampling with conventional histology may lead to underclassification of metastases in cases for which the maximum diameter of the tumor is not sampled on glass slides. A simplified diagram of a tangentially sectioned metastasis (pink) within surrounding LN tissue (gray) is shown (center). In this example, the 2D section under-represents the largest dimension of metastasis and could lead pathologists to underclassify the metastasis with implications on patient treatment.

Although LN staging is one of the most critical components of breast-conserving or breast-removal surgery, the method used for evaluating metastases (conventional histology) is subject to sampling limitations that may lead to underclassification. 2D histology only visualizes a few thin (∼5  μm thick) sections of each LN specimen (typically ∼0.5  cm in diameter), in which those thin sections are typically acquired at a spacing (sampling interval) of only a few tens of microns. Collectively, these tissue sections represent <1% of the specimen. In many cases, histology slides will show cross sections of tumor deposits at a tangential plane and may under-represent the tumor’s largest dimension as shown in Fig. 1.

These limitations motivate the need for a more comprehensive and accurate method of staging LN metastases. There have been efforts to serially section LN metastases for histologic evaluation.^3^^,^^4^ However, generating and digitally aligning hundreds of glass slides per specimen is a labor- and time-intensive process that is not ideal for clinical settings. In recent years, open-top light-sheet (OTLS) microscopy has been developed as a method to rapidly visualize optically cleared specimens in three dimensions.^5^^–^^7^ It enables convenient sample mounting and rapid camera-based acquisition, which improves speed and ease-of-use in comparison to alternative 3D imaging approaches, such as confocal microscopy or traditional light-sheet fluorescence microscopy (LSFM) architectures.^8^^–^^13^ In addition, OTLS is nondestructive to the tissue, permitting downstream histopathology (e.g., immunohistochemistry) and molecular analyses that are becoming increasingly important in breast oncology.^2^

In a typical pathology workflow, pathologists rapidly examine large tissue areas at low resolution before reviewing suspicious regions of interest (ROIs) at high resolution to make a definitive diagnosis. This multiresolution workflow is essential to examine large tissue areas within reasonable time frames. With 3D microscopy, the trade-off between resolution and imaging/interpretation time is even more severe (scaling with the third power rather than quadratically for 2D microscopy). To facilitate efficient imaging workflows, we have developed multiresolution OTLS microscopy, where whole LNs may be rapidly imaged at low resolution before identifying suspicious ROIs to selectively image and diagnose at high resolution.^7^

Although multiresolution OTLS microscopy enables multiscale 3D pathology of large clinical specimens, previously demonstrated pre- and post-imaging workflows (tissue and data processing, respectively) impose limitations that hamper clinical translation. For example, previously reported tissue processing methods are ineffective for certain organs such as lipid-rich LNs (Sec. 2.2). In addition, methods for handling large OTLS datasets impose days to weeks of postprocessing time before pathologists may interpret the datasets, hindering adoption in time-constrained clinical settings. In this report, we overcome several of these limitations, developing a comprehensive multiscale 3D pathology workflow for staging whole LNs. This includes a tissue staining/clearing method for human LNs, multiscale volumetric imaging with OTLS microscopy, and on-the-fly false-coloring for immediate viewing of H&E-like datasets in 3D.

First, to generate high-quality 3D pathology datasets, we developed an optimized method to stain and optically clear human LN specimens using a fluorescent analog of H&E with CUBIC-HistoVIsion (CUBIC-HV) and tetrahydrofuran-based (THF) permeabilization.^14^^,^^15^ This overcomes the staining penetration/uniformity limitations of previously reported methods,^16^^,^^17^ thus enabling deep 3D imaging of whole LNs. In addition, we have developed an imaging and computational workflow that enables pathologists to view false-colored datasets (which mimic H&E staining) in 3D immediately after imaging LN specimens with OTLS microscopy. This workflow achieves a 50× reduction in postprocessing time compared with our prior reports,^12^^,^^14^ improving feasibility of 3D pathology with OTLS microscopy in routine clinical settings. In this workflow, LN specimens are rapidly imaged at low resolution and false colored on-the-fly so that pathologists can quickly view the datasets in 3D to identify suspicious or abnormal ROIs. These ROIs are then imaged at high resolution so pathologists may definitively diagnose each lesion (as a metastasis or benign tissue) and classify metastases as ITCs, micrometastases, or macrometastases. We provide examples in whole LNs for which metastases are diagnosed and classified using our 3D pathology workflow. To evaluate whether 3D pathology may improve staging accuracy in comparison to conventional histology, we compare our 3D pathology results to 2D images that mimic conventional histology. In addition to quantifying the extent to which simulated 2D histology underestimates the size of metastatic lesions, we highlight two examples for which 3D pathology upstages the results of simulated 2D histology, which could have a significant impact on clinical management.

## 2 Materials and Methods

### 2.1 Initial Tissue Preparation

Formalin-fixed paraffin-embedded (FFPE) LN tissues were obtained from breast cancer patients previously treated at the University of Washington Medical Center (UWMC). All specimens were de-identified and transferred to researchers by the NorthWest Biospecimen tissue-bank resource at the UWMC with IRB approval from the UW Human Subjects Division. Per standard clinical practice, all LNs were bisected or cut into thick slices (bread loafs) such that the maximum thickness of each LN specimen was 2 mm. All specimens (bread-loaf slices) from each LN were imaged to enable comprehensive examination of whole LNs. Archived FFPE tissue blocks were de-paraffinized by incubating them at 70°C for 1 h and then immersing them in xylene at 65°C for 48 h.

### 2.2 Fluorescent Analog of H&E Enabled by CUBIC-HistoVision

**Fig. 2:**
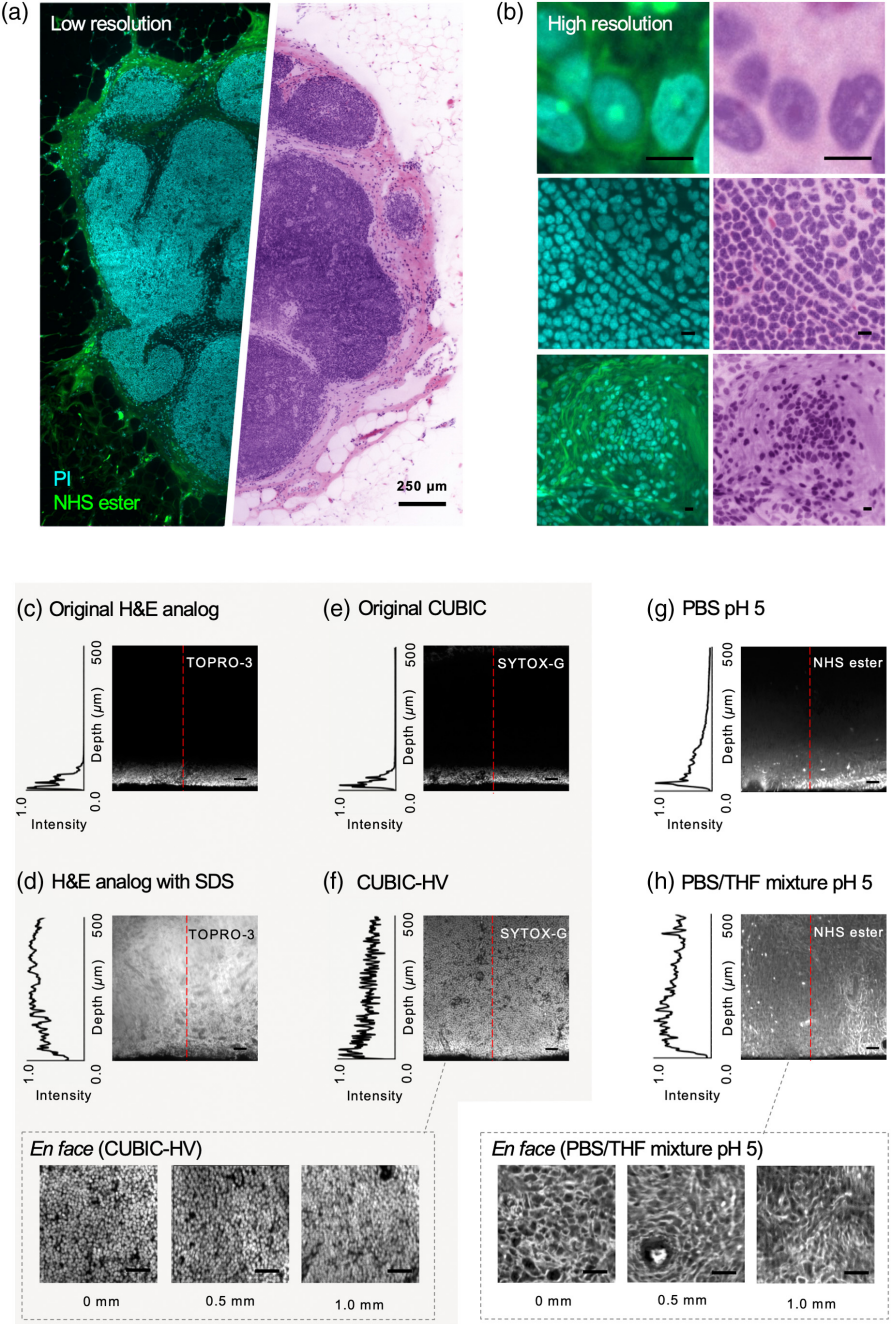
(a) *En face* visualization of an LN stained with our H&E fluorescent analog, cleared with CUBIC-R+(N), and imaged with multiresolution OTLS microscopy. Standard fluorescence intensity visualization is shown on the left at low resolution. H&E-like visualization is shown on the right, which is generated with an open-source false-coloring code that operates on two-channel fluorescence images of tissues stained with a nuclear and cytoplasmic fluorophore.^24^ (b) ROIs shown at high resolution. Standard fluorescence and H&E-like visualizations are shown on the left and right, respectively. Scale bars represent 10  μm. Comparison of various techniques for [(c)–(f)] nuclear and [(g), (h)] cytoplasmic staining in LN tissue. Line profiles of staining intensity as a function of depth in the tissue are shown to the left of each vertical cross-section image. For nuclear staining, we show (c) our original H&E-analog staining protocol, (d) a SWITCH-mediated version of our H&E-analog staining protocol, (e) the original CUBIC protocol, and (f) our final protocol based on CUBIC-HV. At the bottom, *en face* views are shown at various tissue depths for the dataset in (f). For cytoplasmic staining, we compare (g) incubating a specimen with our cytoplasmic stain (AlexaFluor NHS ester) in PBS at pH 5, and (h) our final protocol, where the specimen is incubated with the same cytoplasmic stain in a PBS/THF mixture at pH 5. At the bottom, *en face* views are shown at various tissue depths for the dataset in (h). Scale bars represent 50  μm for vertical cross sections and 25  μm for *en face* views.

A number of methods have been reported for staining fresh and fixed tissues with a fluorescent analog of H&E.^15^^,^^16^^,^^18^ While these reports utilize small-molecule fluorescent dyes, which are known for rapid and efficient penetration into tissue, the density of lipid-rich membranes in LN tissue^19^ impedes penetration and makes it challenging to achieve bright and uniform H&E-analog staining. Here, we describe a protocol that combines CUBIC-HV and THF permeabilization for staining thick LN tissues with a fluorescent analog of H&E and optical clearing with CUBIC-R+(N).^14^^,^^15^ This method enables bright and uniform labeling of LN tissues with a nuclear stain by (1) aggressively delipidating the tissue and (2) moderating ionic interactions between the tissue and the positively charged nuclear stain, which allows for more uniform staining as a function of depth. To achieve a bright and uniform cytoplasmic stain (eosin analog), as reported in Ref. 15, we found that incubating AlexaFluor 647 NHS ester in an aqueous/tetrahydrofuran (THF) mixture at a pH of 5 improved penetration in comparison to adjusting buffer pH alone. We showcase the results of this fluorescent H&E analog in Figs. 2(a) and 2(b), and we describe our fully optimized protocol in the next paragraph.

In our fully optimized protocol, LN tissues are first delipidated with CUBIC-L for 3 days. After subsequently washing the tissues in PBS, the tissues are stained with 2.5  μM SYTOX-G in Sca*l*eCUBIC-1A with 500 mM NaCl at 37°C for 3 days. Tissues are then incubated with 0.5  μg/mL AlexaFluor 647 NHS ester in an aqueous/THF mixture (100 mg/mL THF in PBS) at pH 5 at 37°C overnight. Finally, after washing in PBS, tissues are incubated in CUBIC-R+(N) for 24 h for index-matching purposes (optical clearing) before imaging with OTLS microscopy.^14^^,^^20^ Note we have also found that the nuclear stain propidium iodide (PI, 60  μg/mL) may also be used as a suitable cost-effective alternative to SYTOX-G.

Compared with previously reported methods for nuclear and cytoplasmic staining, we demonstrate that our staining/clearing protocol achieves improved penetration and uniformity in human LNs, both for nuclear labeling [Figs. 2(c)–2(f)] and cytoplasmic labeling [Figs. 2(g)– and 2(h)]. For nuclear labeling, we compare our method to our original H&E-analog staining protocol, in which LN tissues are incubated with 1  μM TO-PRO-3 in 70% ethanol pH4 (30% deionized water) for 48 h.^7^^,^^21^ This protocol achieves poor nuclear staining uniformity as a function of depth [Fig. 2(c)]. We also compare our method to a SWITCH-mediated version of our original H&E-analog protocol, where tissues are incubated in the same buffer as in Fig. 2(c), but with the addition of 10-mM sodium dodecyl sulfate (SDS) for the first 24 h of incubation at 37°C to inhibit binding and allow TO-PRO-3 to diffuse more readily into the tissue (SWITCH “OFF”). The tissues are then incubated in the staining buffer without SDS for 24 h at 37°C to allow TO-PRO-3 to bind to its nucleic-acid targets at depth within the tissue (SWITCH “ON”).^17^ While this method greatly improves penetration of the nuclear stain as a function of depth, SDS compromises the integrity of the nuclear stain and increases background fluorescence [Fig. 2(d)]. Next, we compare our method to the original CUBIC protocol. In this protocol, tissues are pretreated with CUBIC-1 for 1 day before incubating them with SYTOX-G (2.5  μM) in CUBIC-1.^22^ Staining uniformity as a function of depth in this case is not ideal [Fig. 2(e)]. Finally, we demonstrate the CUBIC-HV-based method that is used in our optimized protocol. For the nuclear channel (SYTOX-G), this method improves staining penetration and uniformity compared with the methods shown in Figs. 2(c) and 2(e), with bright and uniform labeling >1  mm deep into tissue [Fig. 2(f)]. For the cytoplasmic channel, we compare the effects of incubating the specimen with AlexaFluor 647 NHS ester in an aqueous/THF mixture compared to staining in an aqueous PBS buffer. In both cases, specimens are incubated with AlexaFluor 647 NHS ester (0.5  μg/mL) in their respective staining buffers (PBS/THF mixture at pH 5 or PBS at pH 5) overnight at 37°C. The addition of THF improves tissue porosity and therefore the diffusion rate,^15^^,^^23^ improving penetration of the NHS ester [Figs. 2(g) and 2(h)].

### 2.3 Multiresolution Imaging with OTLS Microscopy

**Fig. 3:**
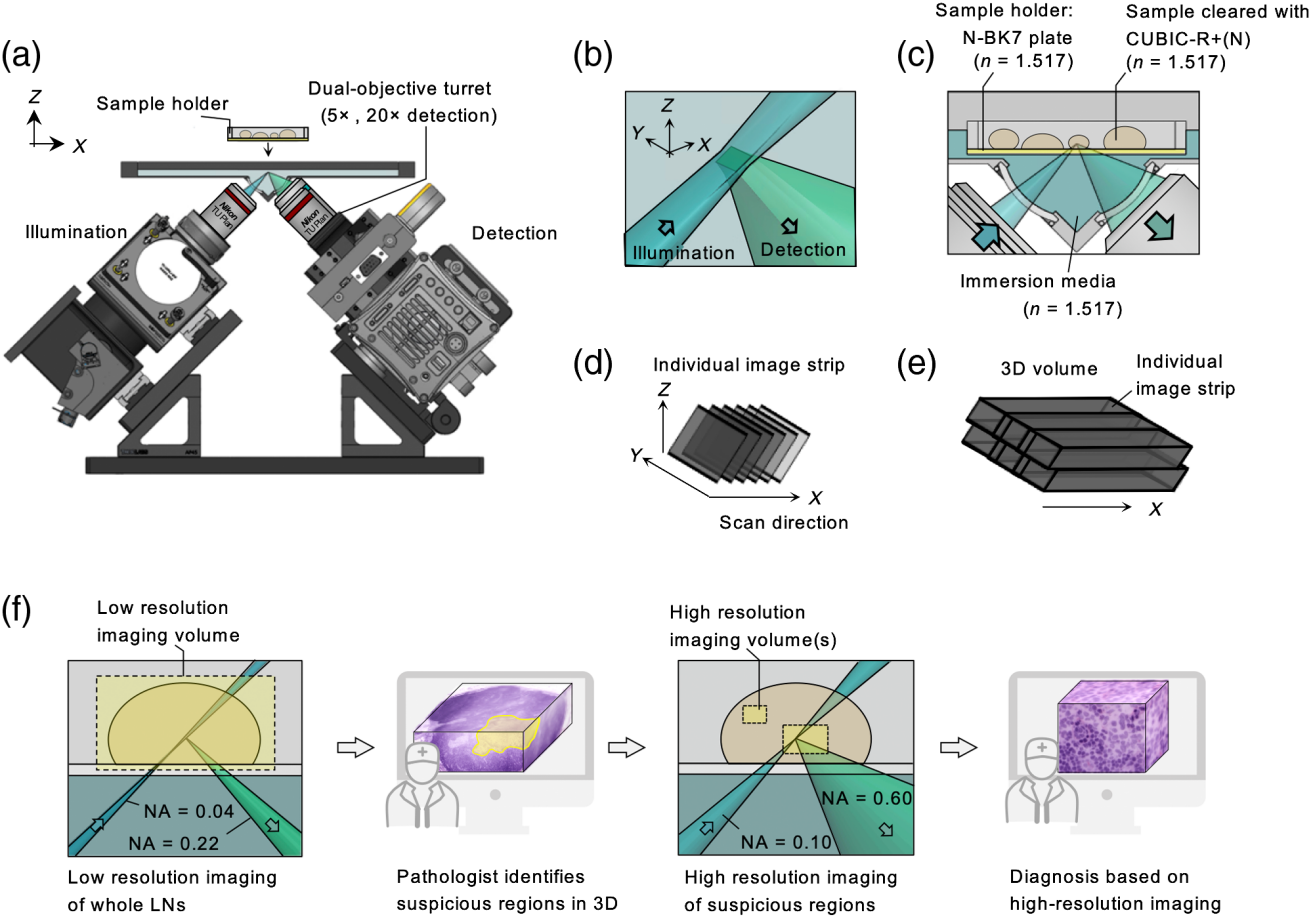
(a) Side-view schematic of the multiresolution OTLS microscope used in this study.^3^ The collection arm is equipped with 5× and 20× objectives on a dual-objective turret for low- and high-resolution imaging, respectively. Specimens may be placed on the modular sample holder, which is attached to a motorized stage (not shown) that translates the specimen in XYZ during imaging. (b) Diagram of the focal region within the specimen. (c) To enable aberration-free imaging, the refractive index of the immersion media, sample holder, and sample must be precisely matched. (d) A single 3D image tile is acquired by stage-scanning the specimen in the X direction. (e) Adjacent image tiles are collected in the lateral (Y) and vertical (Z) directions, which are assembled to enable the visualization of a large 3D volume. (f) Diagram of the pathology workflow used for staging axillary LNs from breast cancer patients. LN specimens are imaged at low resolution, false colored to mimic H&E histology, and are viewed by a pathologist in 3D to identify suspicious ROIs (i.e., possible metastases). These localized regions are subsequently imaged at high resolution in 3D, false-colored, and displayed to a pathologist for definitive diagnosis (tumor versus benign).

After H&E-analog staining and CUBIC-R+(N) optical clearing, LNs were imaged at low resolution (5× collection objective) with a recently reported multiresolution OTLS microscope (shown in Fig. 3).^7^ Laser wavelengths of 488 nm and 660 nm were used when detecting fluorescence of SYTOX-G (hematoxylin analog) and AlexaFluor 647 NHS ester (eosin analog), respectively. Bandpass filters (Semrock FF01-496/LP-25 and Semrock LP02-664RU-25) were used for fluorescence detection of SYTOX-G and AlexaFluor 647 NHS ester, respectively. As shown in Fig. 3 and as previously described for this OTLS system, the refractive index of the sample, sample holder (N-BK7), and immersion medium must be precisely matched for aberration-free imaging. The sample holder is a 0.2-mm-thick substrate made of N-BK7 (n=1.517, Edmund Optics #66-190). The refractive index of the sample’s clearing media is tuned to that of the sample plate (n=1.517) by adding 0.75 mL of water to 50 mL of CUBIC-R+(N). For the immersion medium, a 4:3 mixture of silicone oil (n=1.555, Shin-Etsu HIVAC F-4) and mineral oil (n=1.467, Sigma-Aldrich CAS 8042-47-5) is used (n=1.517).

LN specimens were placed on the flat sample plate and imaged at a rate of $10\;{\mathrm{mm}}^{3}/\min$ at low resolution (5× objective).^7^ After low-resolution imaging with our optimized pipeline (described in Sec. 2.4), the false-colored datasets were viewed in 3D using BigStitcher (an open-source software package in Fiji for efficient visualization of 3D datasets^26^^,^^27^) to identify suspicious ROIs. These regions were then imaged at high resolution (20× objective) at a rate of $0.03\;{\mathrm{mm}}^{3}/\min$ (with B3D compression for high-resolution imaging^27^), false-colored in postprocessing, and shown to a pathologist for definitive diagnosis of localized regions in 3D. With our multiresolution OTLS system, a standard spherically shaped LN with a diameter of 5 mm (volume $∼65\;{\mathrm{mm}}^{3}$) can be imaged at low resolution in ∼6.5  min, whereas high-resolution imaging of such a large volume would require >8  h and would generate ∼80× more data for downstream processing/analysis.

### 2.4 Imaging and Data-Processing Workflow Enables Visualization of H&E-Like Datasets in 3D

**Fig. 4:**
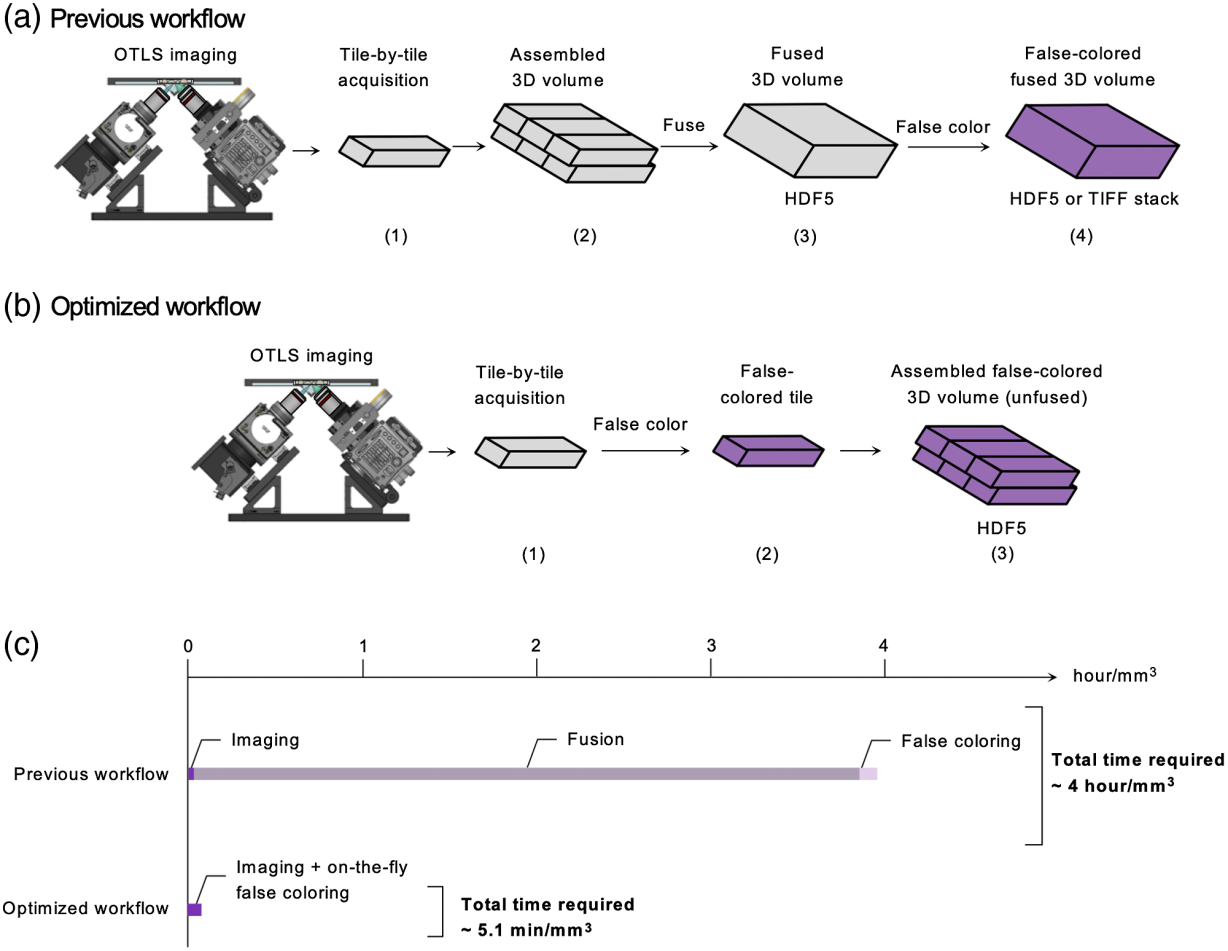
(a) Step-by-step illustration of our previously reported workflow^7^^,^^16^ for OTLS image acquisition, postprocessing, and viewing of false-colored datasets in 3D. A series of volumetric image tiles were (1) acquired sequentially during imaging and (2) assembled to create a large volumetric image of the specimen. To create a “seamless” 3D dataset, the image tiles were then (3) fused into a single 3D image volume. For visual interpretation, the fused datasets could subsequently be (4) false-colored and saved as a stack of RGB TIFFs (or HDF5). TIFF stacks could then be loaded into Fiji for 3D visualization. (b) The workflow we report here enables H&E-like visualization of 3D image data immediately after imaging. After each 3D image tile is acquired (1), it is (2) immediately false-colored while the raw data are in RAM (before the next tile is imaged) and (3) saved at multiple levels of downsampling in a hierarchical data format (HDF5). After imaging is complete, the volumetric false-colored dataset (hierarchical RGB dataset) may be viewed in BigStitcher with an H&E-like appearance. (c) This new workflow reduces postprocessing time by 50× compared to our previous workflow. The time required for each processing step is shown for the previous workflow (top) and optimized workflow (bottom). Here, the reported processing times are for a low-resolution dataset using a workstation equipped with an Intel Xeon processor, NVIDIA TITAN Xp graphics card with CUDA 10.2, and 128 GB of RAM. The time required for false coloring assumes that the depth direction (z axis) is binned by 4× in all cases.

As described in Ref. 7, a volumetric image of the specimen is created by stage-scanning the sample through the light sheet in three dimensions to create a series of 3D image tiles that are stored in a hierarchical data format (HDF5). In the postprocessing workflow used in our prior studies [Fig. 4(a)], the datasets first needed to be fused. Fusion is a processing step performed in BigStitcher^26^^,^^27^ that blends the seams between overlapping tiles and combines the individual tiles into a contiguous and “seamless” 3D volume. After fusion, the two-channel datasets (nuclear and cytoplasmic channels) were false-colored in Python to render an H&E-like appearance.^24^ False-colored datasets were then saved as a stack of 2D RGB TIFF images, which could be visualized in Fiji after loading the image stack.^25^ This workflow (particularly the fusion step) was extremely time consuming and RAM-intensive, requiring $4\;h/{\mathrm{mm}}^{3}$ for low-resolution datasets ($3.8\;h/{\mathrm{mm}}^{3}$ for fusion and $6.5\;\min/{\mathrm{mm}}^{3}$ for false-coloring) on a workstation with an Intel Xeon processor (E5-1620 v4 3.5GHz 4 core), NVIDIA TITAN Xp graphics card, and 128 GB of RAM. Unfortunately, this made it impractical for pathologists to identify suspicious ROIs from low-resolution datasets within reasonable time frames (while the specimen was still mounted on the OTLS system) for subsequent high-resolution imaging, which hindered feasibility for high-throughput clinical applications.

The workflow we report here bypasses image fusion entirely and false-colors individual 3D tiles on-the-fly as they are acquired [Fig. 4(b)]. In other words, for each volumetric image tile that is acquired, the camera frames are streamed to RAM as individual 3D tiles and immediately false-colored, resulting in a color (RGB) image tile. The false-colored 3D image tiles are then stored in a HDF5 data container at full resolution and as multiple downsampled versions (2×,4×, and 8×). The associated metadata (position of each tile according to known stage coordinates, sampling intervals, etc.) are also saved in an XML file. After imaging is completed, the datasets are immediately viewable with an H&E-like appearance in BigStitcher, which assembles image tiles in appropriate positions relative to adjacent tiles according to the coordinates in the XML metadata.^26^ The HDF5 and XML formats used here are similar to those used in previous reports^14^^,^^20^ with the exception that three 8-bit color channels (RGB) are saved (for the H&E-false-colored datasets) instead of the two original fluorescence channels (nuclear and cytoplasmic stains). Total imaging times with this new workflow, including on-the-fly false coloring, is $∼5.1\;{\min/\mathrm{mm}}^{3}$. Note that our false-coloring algorithm “normalizes” the fluorescence intensities, which degrade slightly as a function of depth, such that the H&E false-colored image outputs are relatively uniform in appearance throughout the 3D datasets. Finally, it should be noted that the use of BigStitcher for visualization of H&E-like images has not been previously reported, as BigStitcher is conventionally used for visualization of standard fluorescence rather than volumetric RGB images. We hope this application will broaden the user base of this versatile open-source 3D visualization platform to clinicians and pathologists.

In summary, our imaging and data-processing pipeline is 50× faster than our previous workflows by omitting fusion, false-coloring individual image tiles on-the-fly (which reduces I/O related latencies associated with false-coloring after the data are saved to disk), and using a specialized hierarchical image-formatting method for false-colored data to enable volumetric visualization of H&E-like datasets in BigStitcher. Because the fusion process is not performed (i.e., overlapping regions in adjacent image tiles are not blended), visual seams may persist between tiles due to slight offsets (subpixel to pixel-level misalignments) between the tiles. However, these minor artifacts do not impede visual interpretation by pathologists. Note that there are also unavoidable artifacts in conventional 2D histology (cracks, folds, bubbles, etc.). To further reduce processing time, image tiles are binned by 4× in the depth direction (z axis) before false-coloring is performed, which results in an effective sampling thickness/interval of ∼4  μm in the depth direction for each *en face* image (similar to the thickness of H&E slides). Overall, the workflow reported here streamlines pathologist evaluation of low-resolution datasets before localized high-resolution imaging, unhindered by postprocessing delays. Code for this workflow is available to readers upon request.

## 3 Results

### 3.1 Lymph Node Staging with 3D Pathology Image Atlas

In standard clinical practice, LN metastases are classified based on the largest dimension of the metastasis. Metastases may be classified as ITCs, micrometastases, or macrometastases. This classification determines pathologic nodal stage (pN0, pN0(i+), pN1(mi), pN1, etc.), where the highest stage reflects the presence of macrometastases and indicates a heavy metastatic nodal burden. This directly informs subsequent treatment decisions; in standard practice, the identification of macrometastases typically results in a decision to perform complete axillary LN dissection (subsequent surgery) to reduce the patient’s tumor burden and to improve survival.^2^^,^^28^^,^^29^

**Fig. 5:**
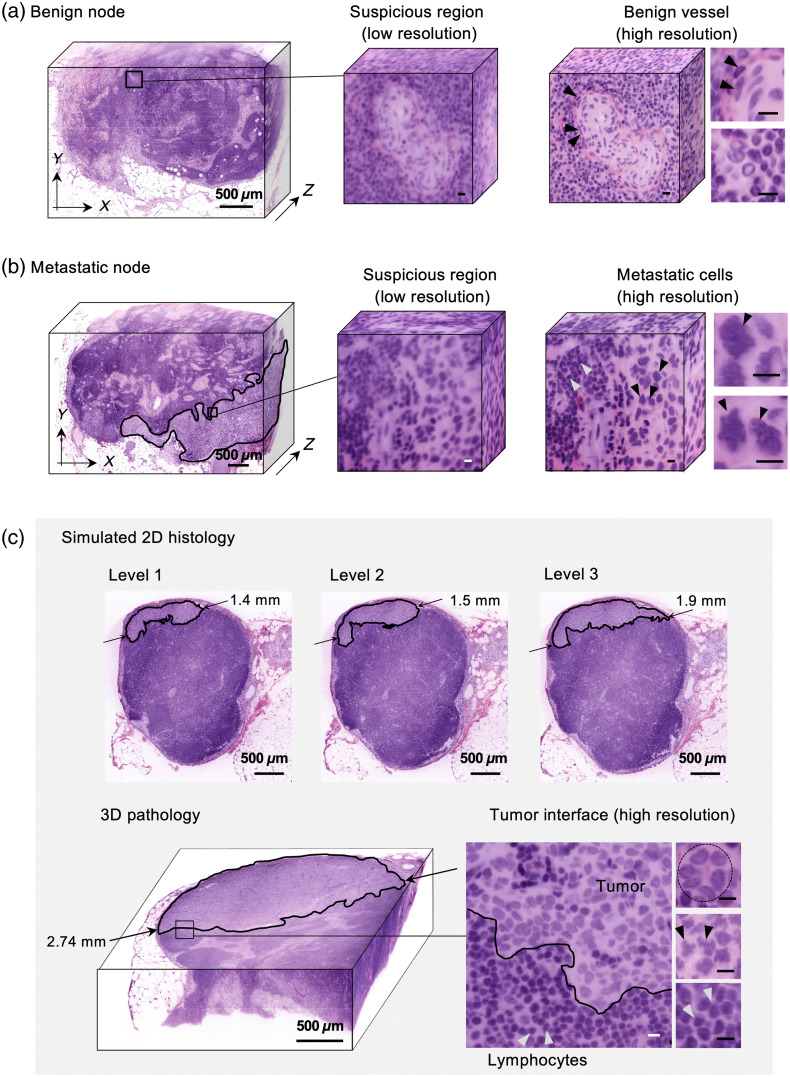
(a) Image atlas of regional LNs classified with our 3D pathology workflow. Low-resolution images of (a) benign node and (b) metastatic node. For both examples, the low-resolution datasets (left) were used to identify suspicious regions (center). Subsequent high-resolution imaging of those localized regions (right) revealed that the suspicious region in panel (a) is a benign vessel, as indicated by the flattened endothelial cells lining the vessel, and that the suspicious region in panel (b) contains cancer cells, as indicated by the enlarged and irregular-shaped nuclei (black arrows). Lymphocytes adjacent to metastatic cells are indicated by white arrows, exhibiting circular darkly stained nuclei that are densely packed (high nuclear-to-cytoplasm ratios). The maximum dimension of the metastatic nodes is used to classify them as individual tumor cells (<200  μm), micrometastases (200  μm to 2 mm), or macrometastases (>2  mm). (c) Top: a micrometastasis (<2  mm) is observed in the simulated histology images. Bottom left: deep 3D imaging reveals that the tumor deposit is a macrometastasis (>2  mm), an upstaging that would lead to a more aggressive treatment plan (complete axillary LN dissection). Bottom right: high-resolution imaging of the interface between the metastasis and benign tissue. ROIs show clustered nuclei that are suggestive of tubule formation (top) and enlarged, irregularly shaped nuclei (middle) that are indicative of cancer. A high-resolution view of benign lymphocytes is also shown (bottom, white arrows). Scale bars of ROIs represent 10  μm.

As was previously described, large LN specimens were deparaffinized, stained with a fluorescent H&E analog (Sec. 2.2), optically cleared, and then imaged at low resolution (Sec. 2.4). False-colored LN datasets (low resolution) were reviewed in 3D in BigStitcher to identify suspicious regions. Suspicious ROIs were then imaged at high resolution, false-colored, and displayed to a pathologist to definitively diagnose the lesion as a tumor metastasis or benign tissue [Figs. 5(a) and 5(b)]. The metastases were then classified as ITCs, micrometastasis, or macrometastasis based on the largest dimension of the metastatic lesion in 3D using Imaris software (Bitplane). Note that there are slight differences in the amount of shrinkage/deformation in thick tissues processed with our labeling/clearing approach versus standard FFPE processing (estimated to be within 5% in this study). Future studies should examine and quantify these differences more carefully and their implications on clinical staging.

### 3.2 Comparison of 3D Pathology to Simulated 2D Histology

The standard procedure for histologic evaluation of regional LNs includes “facing” into the FFPE blocks (cutting into the tissue block to expose a large surface area of tissue) and then obtaining tissue sections at three different “levels” separated by ∼80  μm, covering a total of ∼240  μm in depth. Using our volumetric datasets, we mimicked this sampling procedure for 20 LNs by extracting a 2D image cross section at a depth of 500  μm from the tissue surface (similar to how histotechnologists “level” into a tissue block with a microtome) and then extracting two more cross sections at 80  μm intervals (i.e., two additional “levels”). The 2D images (three levels per LN) were reviewed by a pathologist to diagnose the LN (malignant versus benign) and classify metastatic lesions. We then compared this to the 3D pathology-based classification for each metastasis. For the 10 nodes that contained metastatic deposits, we found that the 2D *en face* images that mimicked conventional histology underestimated the maximum dimension of the tumor deposits by 19% on average (Table 1). These results are summarized in Table 1. In addition, we found that 3D pathology would have led to an upstaging of the LN metastasis in two cases (compared to simulated 2D histology). In both cases, a micrometastasis was observed in the simulated 2D histology images but a macrometastasis (>2  mm maximum dimension) was evident in the 3D pathology datasets as a result of increased sampling of the specimens. One of these examples is shown in Fig. 5(c).

**Table 1:** The maximum dimension of 10 metastatic LNs was determined by simulated 2D histology and 3D pathology, and the results were compared. On average, simulated 2D histology underestimated the maximum dimension of the metastases by 19%. In addition, nodes 5 and 8 are examples for which 3D pathology upstaged the diagnosis from a micrometastasis (based on simulated 2D histology) to a macrometastasis (>2  mm).

Note ID	1	2	3	4	5	6	7	8	9	10
Max. dimension based on simulated 2D histology (mm)	4.7	5.5	3.4	6.7	1.9	3.1	5.1	0.3	2.5	6.1
Max. dimension based on 3D pathology (mm)	5.2	6.0	3.5	6.8	2.7	4.0	5.2	2.2	2.8	7.3
% underestimation of max. dimension with 2D histology	9.6	8.0	4.0	1.4	30.3	22.7	2.3	85.4	10.4	15.9

## 4 Discussion

One of the most critical components of breast-conserving or breast-removal surgery is staging of LN metastases. The status of regional nodes, or degree of metastatic involvement in regional nodes, directly informs subsequent surgical procedures (axillary LN dissection) and downstream treatment decisions. Unfortunately, the standard of care for staging regional nodes, conventional histology, relies on sparse sampling of the tissue via a few thin tissue sections mounted on glass slides. This 2D-based method is particularly prone to sampling errors because the principal feature for assessing LN metastases (maximum dimension) changes with depth in the specimen. In this report, we demonstrate a comprehensive multiresolution 3D pathology workflow that enables assessment of whole regional LNs in 3D and overcomes limitations of standard-of-care 2D histology. We showcase a fluorescent analog of H&E for staining and clearing of human LNs based on CUBIC-HV, overcoming inadequate staining penetration and uniformity seen with alternative staining/clearing techniques. In addition, we demonstrate a new image-acquisition and false-coloring pipeline that enables pathologists to view H&E-like images of LN tissues interactively in 3D immediately after low-resolution imaging. This allows LNs to be rapidly screened *in toto* at low resolution to identify suspicious regions in 3D. These localized regions may then be imaged at high resolution in 3D, false-colored, and subsequently displayed to the pathologist to facilitate definitive diagnosis and classification of nodal metastases. This nondestructive 3D pathology method additionally permits downstream molecular assays after OTLS imaging.

Our multiresolution imaging and data/image-processing processes have been accelerated to enable time- and data-efficient clinical workflows. However, as described in this report, our tissue-staining/clearing protocols, though effective, currently require multiple days. However, this process is largely labor-free and easily automated in a clinical laboratory setting, in which specimens would incubate in specific reagents for hours at a time. Imaging times are important to accelerate so that the imaging device (an OTLS microscope in this case) is not a bottleneck for clinical labs. Likewise, data-processing steps and especially image-interpretation times by pathologists should be minimized because these are costly to scale (i.e., data storage, computational power, and clinician labor). Furthermore, accelerating data/image postprocessing times allows pathologists to screen low-resolution H&E-like datasets rapidly (while the specimen is still on the microscope stage) to identify suspicious regions for subsequent high-resolution imaging without having to unmount and remount specimens, which would necessitate complex coordinate-registration and coregistration steps for multiresolution imaging. In the future, artificial intelligence methods, especially for low-resolution screening of specimens, should be explored to further facilitate rapid LN assessments. Rapid tissue staining/clearing methods may also be possible, as others have been reporting for preclinical research applications.^30^

While our study focused on developing a technical workflow for time- and data-efficient 3D pathology of LN specimens, along with a preliminary feasibility study, a number of larger clinical studies are needed to demonstrate value for patient care. For example, studies should be performed to quantify the sensitivity and specificity of tumor detection using both the low-resolution and high-resolution imaging modes, and optimizing these respective magnification/resolution levels to achieve an ideal trade-off between accuracy and speed for both low-resolution screening of whole LNs and high-resolution definitive diagnosis of suspicious lesions. The ideal multiresolution workflow should ultimately achieve a high overall accuracy for identifying and classifying LN metastases within a time frame that is “acceptable” to patients, clinicians, and those involved in the economics of healthcare. Furthermore, our multiresolution 3D pathology workflow should be compared prospectively with true standard-of-care histology practice (rather than simulated 2D histology images in our study) to demonstrate clinical value. While a retrospective analysis of archived tissues would also be of value, there are a number of obstacles. For example, the original histology reports based on the archived specimens used in our study grouped the analysis of multiple LNs into one diagnosis per patient, making it difficult to compare 2D versus 3D pathology on a per-LN basis. Furthermore, since almost all archived LN tissues are partially consumed for standard histology, subsequent 3D pathology datasets are significantly limited in terms of sampling extent. Future prospective studies comparing 3D versus 2D pathology should ideally perform nondestructive 3D pathology on whole LN specimens prior to performing standard-of-care slide-based (destructive) histology of those same specimens.

## 5 Conclusions

In summary, our nondestructive 3D pathology workflow enables whole human LNs to be examined, with the potential to classify metastatic lesions more accurately than conventional histology. This could have a large impact on patient management and outcomes for patients with breast cancer and potentially many other forms of cancer as well.

## Code, Data, and Materials Availability

Deidentified 3D pathology data for LNs from breast cancer patients may be made available under a material transfer agreement. Instrumentation code, schematics, and image processing codes may be made available from the authors upon request.
